# Severe Hyperandrogenism in 46,XX Congenital Adrenal Hyperplasia: Molecular Physiopathology, Late Diagnoses, and Personalized Management

**DOI:** 10.3390/ijms252111779

**Published:** 2024-11-02

**Authors:** Gianluca Cera, Andrea Corsello, Roberto Novizio, Vincenzo Di Donna, Pietro Locantore, Rosa Maria Paragliola

**Affiliations:** 1 Unit of Endocrinology, Department of Translational Medicine and Surgery, Università Cattolica del Sacro Cuore, Fondazione Policlinico “A. Gemelli” IRCCS, I- 00168 Rome, Italy; gianluca.cera@outlook.com (G.C.); robertonovizio@gmail.com (R.N.); dottdido@libero.it (V.D.D.); pietro.locantore@icloud.com (P.L.); 2 Unit of Endocrine Surgery, Ospedale Isola Tiberina—Gemelli Isola, I-00186 Rome, Italy; andrea.corsello@fbf-isola.it; 3 Departmental Faculty of Medicine, Unicamillus-Saint Camillus International University of Health Sciences, I-00131 Rome, Italy

**Keywords:** congenital adrenal hyperplasia, 21-OH deficiency, 46,XX disorders of sex development, gender dysphoria, sex assignment at birth, sex reassignment

## Abstract

Congenital Adrenal Hyperplasia (CAH) is a group of autosomal recessive endocrine disorders characterized by alteration in adrenal hormonal secretions. The most common form is caused by CYP21A2 mutations that result in 21-hydroxylase deficiency. Clinical features can vary, from salt-wasting forms, characterized by a lack of mineralocorticoid activity with a risk of perinatal-onset adrenal crises, to “simple-virilizing” forms with sufficient aldosterone secretion, up to milder “non-classical” forms, with a variable grade of hyperandrogenism but no severe hormonal deficiencies. During pregnancy, CAH 46,XX fetuses are exposed to elevated androgen levels, leading to a variable grade of virilization and potential central nervous system effects if untreated. These patients are usually (but not always) assigned female at birth, but some cases may be misdiagnosed and assigned male, potentially inducing fertility, gender identity, and sexual behavior issues in adulthood. In these patients, the benefits and risks of a late gender transition should be carefully evaluated. In this paper, we reviewed the literature concerning the most interesting peculiarities of these conditions.

## 1. Introduction

**Figure 1:**
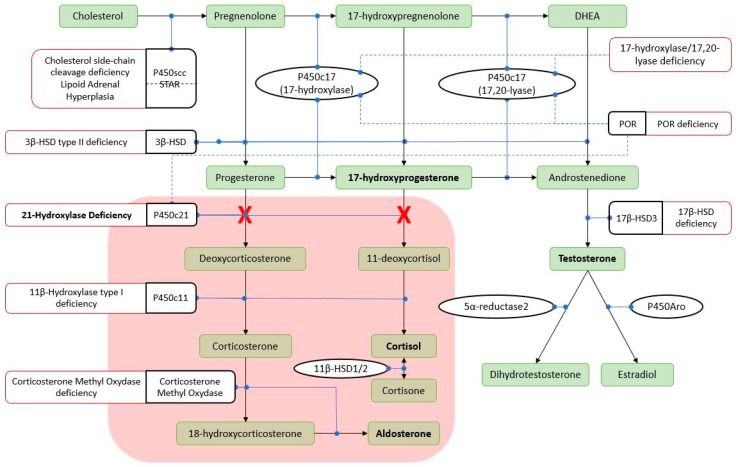
Steroid hormone synthesis pathway. P450scc: cholesterol side-chain cleavage enzyme; STAR: steroidogenic acute regulatory protein; POR: cytochrome P450 reductase; P450c17: steroid 17 alpha-hydroxylase/17,20 lyase; HSD: hydroxysteroid dehydrogenase; P450c21: 21-hydroxylase; P450c11: 11 β-hydroxylase; P450Aro: aromatase. Solid black arrows: conversion; solid blue circles: catalysis; dashed blue lines: stimulation; solid dashed red lines: associated disorders; red box and crosses: hormonal deficiencies in 21-OHD.

Congenital Adrenal Hyperplasia (CAH) is a group of autosomal recessive endocrine disorders caused by genetic mutations affecting one or more steps of adrenal steroidogenesis, leading to cortisol deficiency and other adrenal hormonal alterations. The most common form is caused by mutations in the *CYP21A2* gene, which encodes for 21-hydroxylase, leading to variable cortisol and aldosterone deficiency and accumulation of 17-OH-progesterone (17-OHP) (Figure 1). The severity of the hormonal deficit can vary, with a considerable genotype–phenotype correlation. The clinical phenotypes can be generally classified into a severe congenital-onset classical form and a milder late-onset non-classical (NC) form. Of the classical 21-OHD patients, 75% show a severe mineralocorticoid deficiency with a risk for adrenal crises and are described as salt-wasting (SW) forms, whereas 25% show adequate mineralocorticoid activity and are defined as simple-virilizing [1].

All forms of 21-OHD are characterized by the shunting of adrenal steroid precursors through the functioning androgen pathway and subsequent variably severe hyperandrogenism. Exposure to excessive adrenal androgens during early fetal life (7th–9th gestational week) can lead to a disruption of normal genital development in 46,XX fetuses, with a variable grade of genital virilization in classical 21-OHD, usually staged according to the Prader scale [2]. Moreover, it has been hypothesized that fetal hyperandrogenism may have an impact on brain structure development, some cognitive abilities (such as memory and executive functioning), and future social and sexual behavior, although current findings are inconsistent [3,4,5].

Early diagnosis is of great importance to identify newborns at risk of adrenal crises and adequately treat cortisol deficiency, but also for appropriate sex assignment at birth. Newborn screening programs rely on the evaluation of basal and/or stimulated 17-OHP levels. Some countries, however, do not implement such programs, potentially leading to higher rates of misdiagnosis and/or late diagnosis [6].

In this review, we explore the clinical scenario of severe hyperandrogenism in 46,XX CAH patients. These patients are usually diagnosed at birth by screening programs or because of the finding of ambiguous genitalia; sometimes, however, they may be assigned male at birth with no further investigations and may present to clinical attention later in life with other clinical complications. The multiple issues that should be considered in these patients are discussed below.

## 2. Congenital Adrenal Hyperplasia: Genetic Features and Pathophysiology

The *CYP21A2* gene, located on chromosome 6 (6p21.3), may be subject to variable genetic alterations due to the complexity of this chromosomal region. More than 200 variants of this enzyme are reported, but 10 of them are responsible for almost 90% of cases reported [7].

The clinical phenotype partially correlates with the residual enzymatic activity of 21-hydroxylase. Less than 5% activity leads to classic CAH (salt-wasting or simple virilizing based on the residual aldosterone synthesis), with a strong genotype–phenotype correlation. Instead, an enzymatic activity of 20–50% manifests as milder, non-classic forms, and the phenotype correlates less with the specific genetic alterations. Moreover, around 10% of CAH patients may present the hypermobility phenotype of Ehlers–Danlos syndrome (hEDS). This coexistence is called CAH-X and is caused by heterozygous deletions of the *TNXB* gene that cause the formation of a chimeric *TNXA*/*TNXB* gene and the impairment of the *CYP21A2* sequence [8]. The genotype–phenotype correlation in the most severe forms has strong clinical implications. More severe genotypes cause higher androgen levels and a higher grade of female fetus virilization. This can lead to severely virilized genitalia at birth and, potentially, to male sex assignment [9]. In fact, several cases of severely virilized 46,XX CAH patients reared as males have been reported in the literature with good physical and mental outcomes.

In the usual fetal development, chromosomal sex determines gonadal sex; subsequently, around the 8–12th gestational weeks [10], the gonads’ hormonal activity determines somatic sex. In particular, the expression of the *SRY* gene in 46,XY individuals drives the differentiation of bipotential gonadal precursors into testes. These glands later produce testosterone and anti-Mullerian hormone (AMH), leading to Wolffian duct development and Mullerian duct regression, respectively. Conversely, in female development, the lack of a Y chromosome leads to female gonadal sex, and the subsequent lack of testosterone and AMH lead to a female phenotypic sex [3].

The virilization of 46,XX fetuses’ genitalia is caused by exposure to excessive adrenal androgen from as early as the 7th gestational week, when adrenal steroidogenesis begins [11]. The enzymatic deficit causes the shunting of 17-OHP to androstenedione and, eventually, to testosterone. The rise in testosterone alters the Wolffian–Müllerian differentiation, leading to a variable grade of genital variations staged according to the Prader scale [2]. The Prader scale ranges from stage 0 (normal female) to stage V (complete masculinization). Stage I corresponds to isolated clitoromegaly; stage II is defined by the presence of posterior labial fusion. Stage III patients show a single perineal urogenital orifice with almost complete labial fusion. Last, stage IV is represented by the presence of a phallic clitoris, urethra-like urogenital sinus at the base of the clitoris, and complete labial fusion (which may be interpreted as a hypovirilization with micropenis and hypospadias).

Other than disorders in sex differentiation, less severe hyperandrogenism in 46,XX CAH patients is associated with reduced fertility, hirsutism, polycystic ovarian syndrome, and the development of a deeper voice, with potential physical and psychological consequences [12].

The placental type 2 11β-hydroxysteroid dehydrogenase (11β-HSD) inactivates hydrocortisone, prednisone, and prednisolone, but not dexamethasone. Therefore, dexamethasone-based prenatal therapy is being used in several centers to lower ACTH and androgen levels in 46,XX fetuses with CAH (or at risk of being affected by classical CAH depending on the parents’ genotype). This therapy can effectively prevent virilization, but its use depends on a prenatal suspicion or diagnosis of CAH, with a variable benefits/risks ratio that should be carefully evaluated [9].

## 3. Missed Diagnoses and Late Referrals

Several case reports describe patients with differences of sex development (DSD) presenting later in life because of missed diagnosis at birth or puberty, and this is still a real-world possibility in clinical practice, albeit rare, even in economically developed countries [13,14,15]. In more remote geographical regions, a lack of knowledge and experience of medical providers, in addition to a lack of awareness of patients and families, can lead to late diagnoses [16].

Sometimes, late-referred patients have a history of testosterone priming therapy, are reared as boys, and develop a male gender identity. Often, no anatomical, functional, behavioral, or aesthetical discomfort is reported. Patients sometimes report satisfactory relationships with no complaints. This is quite remarkable, as anatomic variations and the absence of ejaculate often represent a source of physical and/or psychological discomfort [15,17,18]. It has been noted, however, that this may be influenced by conformity bias [19].

Given the social and familial background of these patients, it is possible that some medical history is omitted during the medical visits because of unavailable documentation, inaccurate recalling, and/or possibly a certain restraint in openly discussing such history, even with a doctor. This underlines the great importance of considering the psychosocial background of patients, especially concerning sexual health, and being able to offer a safe and appropriate setting for medical history and physical examination [17].

### Clinical Presentation of Late Diagnoses and Possible Differential Diagnosis

In these late-diagnosed cases, the patient may come to medical attention because of differences in sex development (DSD) and ambiguous genitalia, pubertal complaints, or even infertility.

Clinical examination can reveal hypostaturalism, male hair distribution, and virilized genitalia (Prader stage IV–V), specifically, clitoromegaly (which can be misinterpreted as micropenis) with hypospadias and hypertrophic fused large labia (which can be misinterpreted as a scrotal sac). There can be no evidence of ostium vaginae. Blood pressure is usually normal or low.

**Figure 2:**
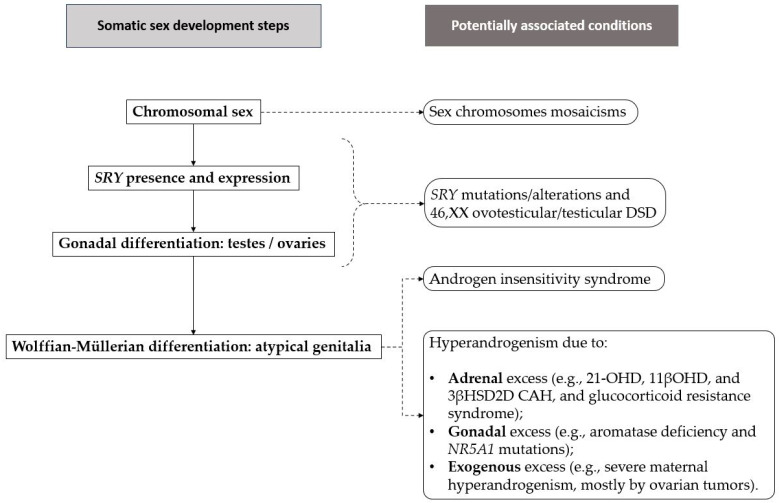
Differential diagnoses in suspected DSD: physiological steps in somatic sex development and potentially associated conditions.

The observation of atypical phenotypic sex appearance with infertility should prompt a suspicion of DSD. Notably, 21-OHD is the most frequent 46,XX DSD [20]. When a DSD is suspected, however, it is necessary to evaluate all possible differential diagnoses of the observed clinical picture, considering alterations in one or more of these three steps (Figure 2) [20,21]:

1. Sex chromosome alterations (mosaicisms).
2. Gonadal differentiation, e.g., *SRY* mutations/translocations.
3. Wolffian–Müllerian differentiation due to altered androgens/AMH secretion or activity.

Hormonal testing of patients presenting with DSD should include basal levels of 17-OH-P, ACTH, cortisol, sodium, potassium, DHEA, androstenedione, anti-Müllerian hormone (AMH), and 11-deoxycortisol, if available, to identify the presence of undiagnosed CAH; in addition, FSH, LH, total testosterone, and estradiol can be measured in undiagnosed adults to evaluate potential gonadal function [22]. Karyotype analysis is indicated for a definitive diagnosis [20]. A pelvic ultrasound and/or MRI should be performed to evaluate the patient’s anatomy.

A diagnosis of 21-OHD can be made in the presence of morning basal 17-OHP levels >10 ng/mL (>30 nmol/L); conversely, it can be excluded with values <2 ng/mL (<6 nmol/L) with a negative predictive value of almost 100%. In the case of 17-OHP levels of 2–10 ng/mL (6–30 nmol/L), it is recommended to carry out a cosyntropin stimulation test; the diagnosis is then confirmed with 17-OHP levels >10 ng/mL (>30 nmol/L) after stimulus [23]. The other hormonal findings are usually compatible with the excess of adrenal androgens (elevated DHEA-S and androstenedione, elevated testosterone, reduced gonadotropins). ACTH-stimulated cortisol can be measured to evaluate the presence of adrenal insufficiency.

## 4. Neonatal Screening

As briefly mentioned, 21-OHD early diagnosis is crucial for the prevention and/or prompt treatment of adrenal crises, as well as for a correct evaluation of sex assignment at birth.

21-OHD can be diagnosed prenatally via chorionic villus sampling and/or amniocentesis and, lately, with cell-free fetal DNA testing from maternal peripheral blood samples. Such prenatal diagnosis is indicated for pregnancies of 21-OHD patients or healthy carriers, as indicated by appropriate genetic counseling [9].

Given the high estimated prevalence of heterozygous *CYP21A2* mutation carriers in the general population (~2%), neonatal screening programs are advised by international guidelines. These programs rely on the detection of basal 17-OH-P levels by immunoassays. In the case of positive results, a new sample is collected and analyzed with better-performing techniques (such as liquid chromatography–tandem mass spectrometry); if no such method is available, ACTH-stimulated 17-OH-P levels should be measured [23].

The results should be appropriately interpreted. 17-OH-P levels are high at birth, especially in males, but they rapidly decrease in normal newborns, whereas they tend to increase in affected children. Moreover, 17-OH-P tends to be higher in premature and/or sick newborns. Finally, 17-OH-P is lowered by prenatal corticosteroid administration (e.g., betamethasone) [23].

The limitations of neonatal screening are the following:

- False positives arising from analytical errors, e.g., cross-reacting substances for immunoassays;
- False positives and false negatives if second-tier confirmation tests are not available;
- False positives and false negatives arising from misinterpretation of the results, e.g., relying on inappropriate reference ranges for gestational age, birth weight, comorbidities, and genetic sex;
- False negatives arising from prenatal corticosteroid administration;
- Missed diagnosis of other CAH forms not characterized by markedly elevated 17-OH-P [15].

In some regions and countries, neonatal screening has not been implemented because of variable cost-effectiveness analysis results. The absence of neonatal screening programs and the rare but possible false negatives may, therefore, lead to missing the diagnosis of 21-OHD patients. This can raise a few fundamental issues. First, a newborn presenting with completely virilized genitalia and no other symptoms may be assigned male with no further evaluation, as reported in this paper and by other authors [24,25]. Second, undiagnosed 21-OHD cases are exposed to a risk of unexpected adrenal crises in the case of salt-wasting forms [23]. Moreover, at puberty, undiagnosed patients may show delayed or absent secondary sexual development [24]. In this setting, it is of note that the first case of CAH ever described in the literature was that of a 46,XX male [26]. Last, untreated or undertreated cisgender patients develop variably severe features of hyperandrogenism. Females may present with hypostaturalism, alopecia, hirsutism, dysmenorrhea, and anovulation, secondary PCOS, genital abnormalities, and possibly a certain grade of masculinization of social and psychosexual features. Males may present with hypostaturalism, alopecia, reduced sperm count, low testosterone, and testicular adrenal rest tumors (TARTs). For all these problems, patients of both sexes may complain about unsatisfactory sexual behavior and/or infertility [27].

All these conditions may lead to late diagnosis and treatment with a risk of severe and/or irreversible events (e.g., potentially lethal adrenal crises, growth, and pubertal abnormalities), so that adequate prenatal diagnosis and neonatal screening programs should be implemented [20]. Moreover, an unthoughtful sex assignment at birth may have an impact on fertility, sexual health, gender identity, and general well-being (see Section 5).

## 5. Treatment of Glucocorticoid Deficiency

In infancy and adult life, classical CAH patients require hormonal replacement therapy with glucocorticoids: hydrocortisone, prednisone, prednisolone, and methylprednisolone are all being used, with hydrocortisone associated with better long-term outcomes [28]. In accordance with data concerning Addison’s Disease, fractionated daily doses of dual-release hydrocortisone can be used, with data showing better control of CAH compared to prednisolone [29]. Prednisolone and methylprednisolone, however, thanks to their longer half-life, can be administered once daily with better compliance. Last, dexamethasone is associated with a more potent reduction in ACTH and androgen levels [10]. While dexamethasone provides the greatest ACTH and androgen suppression, it is associated with more adverse effects, such as lower bone mineral density and higher BMI [28]. Interestingly, a recent case series of Indonesian 46,XX CAH patients who refused glucocorticoid therapy showed adequate virilization without episodes of severe illness and hospitalization. The authors do not advise such a choice because the risk of adrenal crises cannot be excluded [19]. Moreover, in the case of patients refusing glucocorticoid therapy, ACTH levels remain persistently elevated, with a possible increased risk of developing adrenal masses with a potential transformation risk [30]. During pregnancy, glucocorticoid therapy should be continued in classical CAH forms and is sometimes used in non-classical patients, but the choice of glucocorticoid and the use of dexamethasone for preventing fetal genital virilization remain debated issues (see above) [9].

## 6. Sex Assignment at Birth

As mentioned above, virilized 46,XX CAH patients are usually diagnosed shortly after birth, at the latest. In these cases, other than starting hormonal replacement therapy for the glucocorticoid and potential mineralocorticoid deficiency, patients should undergo comprehensive evaluations for appropriate sex assignment and follow different therapeutic strategies based on the chosen approach. Apart from appropriate glucocorticoid and/or mineralocorticoid replacement, assigned-female patients may undergo feminization surgery; conversely, assigned-male patients will need testosterone replacement therapy. Usually, 46,XX CAH patients are assigned female at birth, even in the case of more severe virilization, with the main scope of preserving future fertility. However, several cases of male assignment have been reported in the last few decades. It appears then that most of these patients were assigned male not because of a proper complete evaluation but rather because of sociocultural pressure, missed diagnosis, unavailability of specialized care, or a combination of these factors. Most of these cases, in fact, belong to one of two categories: (1) early-diagnosed children with non-acceptance of a female assignment by the family, and (2) late-diagnosed children presenting with late salt-losing crises or pubertal disorders in adolescence, at the latest [24].

The process of sex assignment requires comprehensive multidisciplinary evaluations with experienced clinicians. Several factors need to be considered [31]: potential for future fertility, surgical options, genital appearance, hormonal replacement therapy, and psychosexual implications of all decisions. Moreover, parents of newborns with atypical genitalia can suffer from variable but considerable distress [21,32,33]: this aspect should be taken into account and lead to a shared decision-making process. It should not be, however, the only factor to be considered, even when the child is born in a more constraining social context [34]. Some authors support the consultation of a religious authority in the case of Muslim patients [35].

Essentially, some authors have advocated for female assignment of 46,XX CAH patients [36,37]. However, other authors recommend a male assignment to be considered, especially in severely virilized patients and/or in the case of late diagnosis, after a comprehensive evaluation of all aspects, including the sociocultural factors at play [38,39]. This led to the current recommendations in the Endocrine Society guidelines, which advocate for female assignment in most cases but recognize controversy and the need for open discussions in the case of male phenotype [23]. In fact, given that no randomized clinical trials can be carried out, observational studies comparing male and female assignment of 46,XX individuals can and should be carried out to guide clinical recommendations [1,40]. The following paragraphs focus on the different aspects taken into account when evaluating gender assignment in these patients.

### 6.1. Potential for Future Fertility

As mentioned above, current guidelines on sex assignment at birth recommend female assignment for most, but not all, 46,XX patients because appropriate glucocorticoid therapy and, if necessary, genital surgery can preserve and/or restore a potential for future fertility [23,40].

Fertility is possible only with female sex assignment. Even in that case, a low fertility rate has been reported for female CAH patients. Several studies have subsequently underlined that the pregnancy rate in adequately treated CAH patients is equal to the general population: other factors, such as psychosexual and behavioral characteristics, could lead these patients to a lower desire for pregnancy and/or lower rates of romantic and sexual relationships [41,42].

In the case of male assignment at birth, the subsequent infertility may bring psychological consequences that should be taken into account, such as a risk for depression, social isolation, and stigma [43].

These aspects should be discussed with the patient’s parents and considered in the sex assignment decision-making process [44].

### 6.2. Surgical Options: Benefits and Harms

Male assignment implies oophorectomy, hysterectomy, and/or treatment of gynecomastia, and/or urethrorrhagia, and infertility [24].

Conversely, female assignment often requires feminizing surgery. Surgical interventions include separation of the sinus urogenitalis, vaginoplasty, remodeling of the labioscrotal folds, clitoridectomy, and/or reduction clitoroplasty (or clitoreduction) [45]. However, high rates of complications and/or unsatisfactory outcomes have been reported [46]. Therefore, the effectiveness and complications of all available surgical options need to be carefully weighed. As randomized clinical trials have not been conducted, no clear evidence-based recommendations have been issued.

Benefits of feminizing surgery include prevention of urinary tract infection and hydrometrocolpos, lower (but not absent) risk of stigmatization for a girl with atypical genitalia, and reduction of parental anxiety. Complications include vaginal stenosis, scarring, urethra–vaginal fistulae, urinary incontinence and infections, and a potential decrease in clitoral sensitivity and sexual well-being [23,46].

One of the most debated aspects of feminizing surgery is the timing of intervention because of clinical, ethical, and sociocultural issues [47]. Late surgery would allow for a more shared decision-making process that could include the individual preferences of the patients, so this has been discussed from an ethical point of view, leading some authors to advocate late surgery [46]. Moreover, clitoromegaly can partially decrease over time with standard medical treatment, thus reducing the need for clitoroplasty. On the other hand, early surgery is preferred retrospectively by most patients; moreover, it is likely that there are better tissue responses to surgery at a younger age, as well as technical advantages in one-stage vagino- and clitoroplasty and a lower psychological distress for the patients [45]. Last, some authors recommend a two-stage surgery with clitoral reduction in infancy and vaginoplasty at puberty, as there is no indication of a vaginoplasty before menarche and puberty [22].

All authors agree on the need for expert and trained surgeons to carry out these procedures to minimize risks and complication rates and optimize outcomes. Moreover, functional outcomes should be the first-sought outcome rather than cosmetic appearance [22,46].

### 6.3. Genital Appearance

The Prader stage of virilization of these patients greatly influences this decision, as it has an impact on all the above factors: whereas I–III Prader Stage patients are usually assigned female, IV–V Prader Stages can be associated with worse feminizing surgical outcomes, higher complications, and are often more difficult to evaluate. In fact, as mentioned above, current guidelines recommend female assignment at birth, but controversy is recognized in the case of Prader V patients [23]. These patients were indeed usually assigned male because of the limitations of genital surgery and for a presumed correlation between genital phenotype and gender identity [48]. Nowadays, these patients tend to be assigned female. However, in some cases, they are indeed assigned female at first but reassigned male after a few weeks upon re-evaluation. Finlayson et al., for example, conducted a multicentric American study including 50 cases of 46,XX CAH, all assigned female at birth, with Prader stage ranging III to V. Two of these patients were reassigned male within the first 2 weeks of life mostly because of their external genitalia phenotype [49].

### 6.4. Psychosexual Considerations

There are psychosexual consequences following every possible decision concerning sex assignment at birth.

Central nervous system exposure to androgen excess during fetal life has a potential impact on gender identity, sexual orientation, and social behavior [3]. However, post-natal endocrine assets, as well as social dynamics, appear to play an important but variable role [50,51,52]. Still, the relevance of each of these factors to each individual may be variable [24].

An individual’s gender identity seems to have a multifactorial origin with a relevant heritability [53]. In CAH patients, a correlation with fetal hyperandrogenism seems to be likely, even if clearly insufficient to explain the variability of gender identity [24]. Gender identity development is remarkably flexible [36], and sociocultural factors have been reported to influence such tract [24,51]. In any case, 46,XX CAH patients with marked genital virilization mostly develop a gender identity corresponding to sex assignment at birth [54]. Nevertheless, a higher rate of gender dysphoria has been reported [40] than in the general population, both in the case of female and male assignments, in accordance with other DSD [50]. Moreover, gender retransition has been described in one case [54]. It is of note that an atypical gender-role behavior does not necessarily translate into transgender identity later in life, underlining that gender-role behavior in childhood and gender identity are two linked but different aspects [36,55]. Other authors, however, have registered “behaviors appropriate for male gender” in young adulthood in about one-third of their cohort of 46,XX assigned-female CAH patients [56]. Interestingly, some authors explored the association between gender dysphoria and several factors, i.e., inadequate treatment, null-genotype, late diagnoses, a higher degree of virilization, type of CAH, or higher levels of androgens. None of these subgroups of patients were found to be associated with a higher risk of gender dysphoria. Thus, no recommendations can be made in favor of female versus male assignment in any of the analyzed subgroups [24]. A recent systematic review on gender dysphoria and DSDs found a higher rate of gender dysphoria in 46,XX CAH patients reared as males compared to those reared as females, thus recommending female sex assignment [37]. In contrast, several case reports and series observed no gender identity disorder and general well-being in patients reared as males [57,58,59]. Thus, as mentioned above, in selected subgroups of patients, i.e., Prader V/late diagnosed CAH, male rearing can be considered and even advisable [39]. Finally, some authors have noted a higher proportion of non-binary gender identity in patients with DSDs, including CAH patients. Whether DSD patients dissatisfied with their sex assignment should be included in the category of gender dysphoria is still a debated issue. This is also influenced by how gender identity is assessed (i.e., a binary male–female system or a bimodal continuum) [22]. Thus, some authors have advocated for longitudinal studies that are adequately designed to the specificity of these patients [60].

As for sexual orientation, great heterogeneity exists on how this aspect is studied and reported in the literature; however, it appears that females with CAH show a higher proportion of homosexual and bisexual orientation compared with the general population, whereas both 46,XY and 46,XX males with CAH only show heterosexual orientation, as far as it has been reported to date [52]. The higher proportion of non-heterosexual orientation in female CAH patients has been linked with hyperandrogenism during fetal brain development, but it is likely that many other factors come into play, such as post-natal hyperandrogenism, consequences of atypical genitalia (e.g., stigma, impaired sexual function, and pleasure after surgery), and a higher fluidity and variability in sexual orientation in females compared to males [3,52].

Other than the psychosocial consequences of gender identity and sexual orientation, many other factors influence mental and sexual health in later life. Altered levels of androgens and/or glucocorticoids have been associated with emotional and behavioral alterations with potential mental health consequences [61,62]. Moreover, fertility and surgery, discussed above, have consequences on real and perceived body image, possibility of satisfactory sexual behavior, etc., with possible mental health consequences in both males [63] and females [64]. In the case of later development of gender dysphoria, feminizing surgery could represent an added negative stressor for the patient’s mental health [24]. Also, the need for frequent genital medical inspection, especially in pre-adolescents, can cause significant discomfort and should be carried out carefully [31]. Last, being affected by a chronic disease can be a stressor by itself [24].

All these aspects can be a source of distress for both male- and female-assigned patients: they can be a cause of stigma, discrimination, social exclusion and isolation, anxiety, depression, and trauma, with an increase in psychiatric disorders and psychological distress, especially in some cultural, familial, and social environments [24,52]. This can strongly affect their mental health, with a need for appropriate medical and/or psychological counseling [17]. Many case reports and case series report patients lost at follow-up, possibly because of psychological and/or social distress of the patient and/or their family [24,63,65].

Nonetheless, several authors have reported the psychosexual well-being of 46,XX assigned-male CAH patients, with no significant distress (e.g., [39,66]).

### 6.5. Sex Hormone Replacement Therapy

Both female and male sex assignments require hormonal replacement therapy with glucocorticoids and/or mineralocorticoids. However, in the case of male assignment, the need for lifelong testosterone replacement therapy must be considered [22].

## 7. Conclusions

We focused our review on 46,XX simple-virilizing 21-OHD patients with severe hyperandrogenism, characterized by a very late diagnosis. The peculiarities of these cases allow us to revise the current pitfalls of CAH diagnosis and advocate for a strongly tailored and personalized approach to the management of these patients.

In transgender care, adult patients usually come to medical attention seeking gender-affirming medical interventions. The role of the clinician is to assess the patient’s ability to understand the effects and consequences of each intervention, to evaluate the mental health needs, and to thoroughly inform the patient so that real consent to treatment can be obtained [67]. Patients with DSDs require different evaluations compared to transgender patients, and gender assignment decisions must consider all the aspects presented above [24]. However, patients’ needs, desires, and demands, as well as exhaustive information, must be at the center of any medical intervention, especially regarding one’s gender identity.

In late-diagnosed patients, a systematic evaluation of the development of gender-related behavior and gender identity should be performed before any decision on definitive gender assignment; however, this is often neglected [34]. Such a late diagnosis should not discourage clinicians from evaluating all these aspects.

In accordance with all the previous considerations, the adequate psychosocial health of these patients favors the confirmation of male gender. These cases can encourage clinicians to rethink gender identity in 46,XX CAH patients, when appropriate, refusing any ideological standpoint and focusing on an individualized approach.
